# Cobalt Content Effect on the Magnetic Properties of Ni_50-x_Co_x_Mn_35.5_In_14.5_ Annealed Ribbons

**DOI:** 10.3390/ma14195497

**Published:** 2021-09-23

**Authors:** Łukasz Dubiel, Andrzej Wal, Ireneusz Stefaniuk, Antoni Żywczak, Piotr Potera, Wojciech Maziarz

**Affiliations:** 1 Department of Physics and Medical Engineering, Faculty of Mathematics and Applied Physics, Rzeszow University of Technology, Powstancow Warszawy 12, 35-959 Rzeszow, Poland; 2 Institute of Physics, College of Natural Sciences, University of Rzeszow, Pigonia 1, 35-959 Rzeszow, Poland; wal@ur.edu.pl (A.W.); ppotera@ur.edu.pl (P.P.); 3 Center of Teaching Technical and Natural Sciences, University of Rzeszow, Pigonia 1, 35-959 Rzeszow, Poland; istef@ur.edu.pl; 4 Academic Centre for Materials and Nanotechnology, AGH University of Science and Technology, A. Mickiewicza 30, 30-059 Krakow, Poland; zywczak@agh.edu.pl; 5 Institute of Metallurgy and Materials Science, Polish Academy of Sciences, Reymonta 25, 30-059 Krakow, Poland; w.maziarz@imim.pl

**Keywords:** Ni-Co-Mn-In ribbons, Heusler alloys, EMR, transformation, annealing

## Abstract

We present a study of the annealing effect and its influence on magnetic and structural properties for a series of Heusler alloys ${\mathrm{Ni}}_{50-x}{\mathrm{Co}}_{x}{\mathrm{Mn}}_{35.5}{\mathrm{In}}_{14.5}$ (x=0,3,5) prepared in ribbon form. We studied the morphology and composition using scanning electron microscopy (SEM) equipped with an X-ray microanalyzer (EDX). The magnetic properties were determined by two methods: electron magnetic resonance (EMR) and vibrating sample magetometer (VSM). We found that cobalt content in the annealed samples reveals an additional magnetic phase transition at lower temperatures.

## 1. Introduction

The Ni-Mn-based alloys are a very attractive group of multifunctional materials due to their unusual properties and potential application in different areas of life, e.g., in medicine, spintronics, or environmentally friendly refrigeration. Moreover, the physical properties of these materials, such as their magnetocaloric properties or magnetic-shape memory, strongly depend on the atomic ordering and the location of atoms in the crystal structure. Even slight changes in lattice site occupation result in noticeable changes in both magnetic and structural properties of these materials. Such modification can be done in two ways, firstly by substitution of appropriate atoms, as in the case of Ni-Co-Mn-In, which was obtained by substitution of Ni atoms in the ternary alloy with Co [1,2,3,4]. The second, simpler method is by applying well-defined heat treatment to the base alloy [5,6]. In the literature, one can find various examples of heat-treating, using Ni-Mn-based alloys, such as annealing with slow cooling or quenching [1,5,7,8]. The heat-treating duration and temperature also vary, but generally, in the ribbons, this time is relatively short compared to bulk samples [9].

The fabrication of ribbons by the melt-spinning method leads to stresses as a result of the rapid solidification process. The annealing process leads to relaxation [10,11], i.e., the removal of such stresses, which are associated with a change in the distance between manganese atoms. This, in turn, may cause changes in the magnetic interactions between these atoms [12], and thus changes in their magnetic properties.

The annealing parameters were chosen on the basis of experiments described in literature [1,5,7,11,13]. The order–disorder transition (ODT), which for the Ni-Mn-In alloy type takes place at 950 K [13], plays a very important role in the selection of the annealing temperature. Typically, annealing is carried out at a temperature greater than 950 K. For example, Recarte et al. [7] report that annealing at such temperatures followed by a slow cooling favors the production of a larger fraction of the $L2_{1}$ structure than rapid cooling. Although, there are also reports [1] that long-term annealing below the ODT temperature also promotes the production of the $L2_{1}$ structure.

Furthermore, there are other annealing methods used in off-stoichiometric Ni-Mn-In and Ni-Mn-Sn alloys [14,15], including annealing in a magnetic field. This procedure leads to the decomposition of the off-stoichiometric alloy into the cubic $L2_{1}$ phase and a second phase with lower symmetry [14,15,16,17], i.e., ferromagnetic ${\mathrm{Ni}}_{50}{\mathrm{Mn}}_{25}X_{25}$, (X=In,Sn), precipitates are embedded in a ${\mathrm{Ni}}_{50}{\mathrm{Mn}}_{50}$ non-magnetic matrix.

In this paper, we investigated annealed off-stoichiometric Ni-Co-Mn-In ribbons with three different compositions. Based on electron magnetic resonance (EMR) spectra and magnetization data, we discussed in detail the magnetic properties of these alloys and their dependence on the cobalt content in the alloy.

## 2. Materials and Methods

Three alloys ${\mathrm{Ni}}_{50-x}{\mathrm{Co}}_{x}{\mathrm{Mn}}_{35.5}{\mathrm{In}}_{14.5}$ (x=0,3,5) were prepared by the melt-spinning method according to the procedure, which was described in references [2]. Before measurements the samples were annealed at 1173 K for 30 min and then slowly cooled in a furnace for 12 h. Samples with different cobalt content were labeled NC0MI, NC3MI, and NC5MI for 0, 3, and 5 at.% of cobalt, respectively.

Morphology and composition of samples were inexamined using Tescan Vega 3 scanning electron microscope (TESCAN ORSAY HOLDING, Brno, Czech Republic) equipped with the EDX Bruker Quantax microanalysis system.

The electron magnetic resonance measurements were performed using a Bruker ELEXYS E580 spectrometer (Karlsruhe, Germany) equipped with the Bruker liquid N gas flow cryostat with the 41131 VT digital controller (Bruker Analytische Messtechnik, Rheinstetten, Germany), within the temperature range 110 K ≤T≤ 450 K. The EMR signal was registered with the following parameters, a 100 kHz modulation and amplitude of 0.1 mT. The microwave power was 23.77 mW for NC0MI, NC5MI and 15.00 mW for NC3MI, respectively. All EMR measurements were performed in the X-band (9.44 GHz). The resonance position was calibrated by DPPH.

The magnetization results were obtained by the use of LakeShore 7407 vibrating sample magnetometer (VSM, Westerville, OH, USA). The field cooled (FC) and the field heated (FH) curves were registered at $\mu_{0}H=0.01$ T and $\mu_{0}H=0.1$ T in the temperature range of 85 K ≤T≤ 450 K.

## 3. Results and Discussion

### 3.1. Morphology and Composition

**Figure 1:**
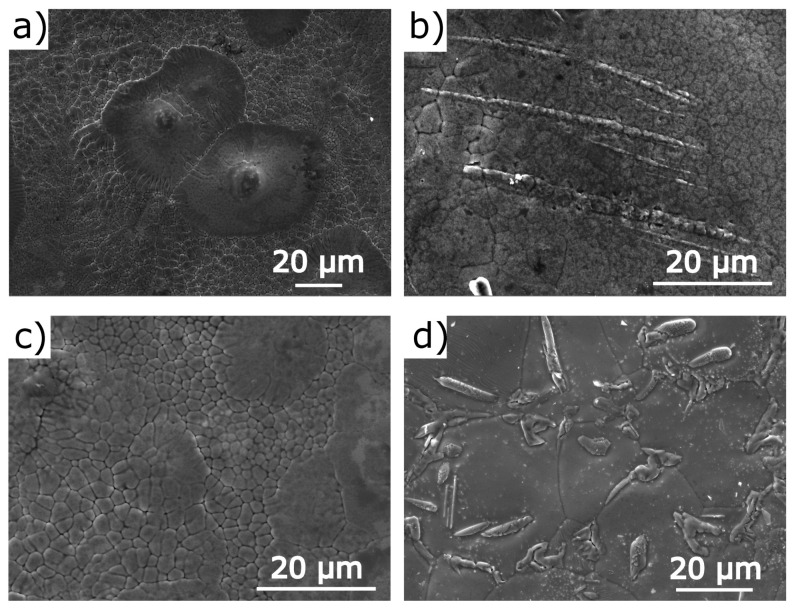
Microstructure of ${\mathrm{Ni}}_{50-x}{\mathrm{Co}}_{x}{\mathrm{Mn}}_{35.5}{\mathrm{In}}_{14.5}$ ribbons at RT: ${\mathrm{Ni}}_{47}{\mathrm{Co}}_{3}{\mathrm{Mn}}_{35.5}{\mathrm{In}}_{14.5}$ as-spun (**a**) and annealed (**b**) ribbons; ${\mathrm{Ni}}_{45}{\mathrm{Co}}_{5}{\mathrm{Mn}}_{35.5}{\mathrm{In}}_{14.5}$ as-spun (**c**) and annealed (**d**) ribbons.

Figure 1 shows the SEM images of surfaces for melt-spun and annealed (1173 K) NC3MI and NC5MI at room temperature (RT). The all melt-spun ribbons (Figure 1a,c) exhibit a granular surface topology. The grain size is not uniform and grains are randomly distributed throughout the area of ribbons. Although the ribbon is obtained from polycrystalline ingots, the columnar crystal directions in the ribbon are preferentially oriented perpendicular to the ribbon plane [18,19].

The surface morphology of the ribbons after annealing has changed significantly. Additionally there is also a difference in the morphology of the annealed ribbons with different cobalt content.

As shown in Figure 1b, the grains in NC3MI become more uniform after annealing and the largest grains have disappeared. In addition to grains, on the surface of the ribbons one can see the second phase in stripes form. EDS analysis showed that the second phase composition differs from the matrix composition by a significant increase in cobalt concentration (11.35 at.%), at the expense of manganese (29.65 at.%) and indium (11.76 at.%). Figure 1d presents the SEM image of the surface of NC5MI. The morphology of this sample is very rich and significantly different from the two previous alloys. First of all, the increase in grain size after annealing is clearly visible. In addition to the second phase in the striped form, one can see the eutectoid form on the surface of this ribbon. Similarly to the NC3MI ribbon, the second phase composition is different from the matrix composition.

### 3.2. Magnetic Properties

**Figure 2:**
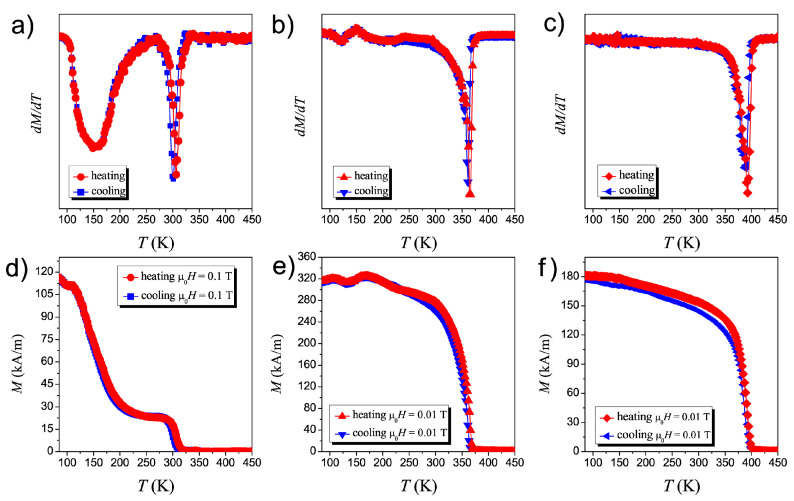
FH and FC magnetization curves as a function of temperature of ${\mathrm{Ni}}_{50-x}{\mathrm{Co}}_{x}{\mathrm{Mn}}_{35.5}{\mathrm{In}}_{14.5}$ annealed ribbons: ${\mathrm{Ni}}_{50}{\mathrm{Mn}}_{35.5}{\mathrm{In}}_{14.5}$ (**d**), ${\mathrm{Ni}}_{47}{\mathrm{Co}}_{3}{\mathrm{Mn}}_{35.5}{\mathrm{In}}_{14.5}$ (**e**) and ${\mathrm{Ni}}_{45}{\mathrm{Co}}_{5}{\mathrm{Mn}}_{35.5}{\mathrm{In}}_{14.5}$ (**f**). The upper panel contains the corresponding dM/dT curves (**a**–**c**).

The magnetic properties of the ${\mathrm{Ni}}_{50-x}{\mathrm{Co}}_{x}{\mathrm{Mn}}_{35.5}{\mathrm{In}}_{14.5}$ ribbons were studied as a function of temperature. The magnetization curves M(T) obtained for the samples with different content of Co are presented in Figure 2. The FH and FC magnetization curves for NC3MI and NC5MI were obtained in a field 0.01 T. For NC0MI, magnetization cures were registered at 0.1 T due to a very weak signal.

The nature of the M(T) curves for annealed ribbons differs significantly from non-annealed ribbons [20,21,22]. The M(T) of the NC5MI sample displayed in Figure 2f is similar to magnetization of the ribbon before annealing [21]. This magnetization curve does not exhibit any thermal hysteresis and has a clear shift toward higher temperatures relative to non-annealed alloy [20,21].

Also, for the two remaining annealed samples, NC0MI and NC3MI, no clear temperature hysteresis is visible, and the behavior of M(T), especially for NC0MI, is unusual. In order to better present phase transitions and to determine precisely the temperatures of these transitions, one should consider the temperature dependence of the derivative of magnetization dM/dT (Figure 2a–c). At high temperature ranges, each sample exhibits a magnetic phase transition from paramagnetism to ferromagnetism with Curie temperatures equal to 300 K, 363 K, and 392 K for NC0MI, NC3MI, and NC5MI, respectively. However, the magnetization below $T_{C}$ suggests that these materials are multi-phased. Especially for NC0MI, there is a visible narrow region when magnetization is saturated, but with the further reduction of temperature, the magnetization starts to increase again around 225 K. Similar magnetization curves were observed for ${\mathrm{Ni}}_{50.3}{\mathrm{Mn}}_{35.3}{\mathrm{In}}_{14.4}$ alloy produced by annealing under a magnetic field [15] and for ${\mathrm{Ni}}_{2.14}{\mathrm{Mn}}_{0.55}{\mathrm{Sb}}_{1.3}$ after homogenization [23]. Considering the derivative of *M* versus *T* (Figure 2a), one can see that at around 150 K the next magnetic ordering temperature occurs. The character of this magnetic phase transition strongly depends on Co content. With increasing of cobalt concentration, the intensity of the low temperature peak in the dM/dT value gradually decreases. For a sample containing 3 at.% of cobalt this peak still exits, but its minimum is much less visible than for NC0MI. In the Figure 2c corresponding to the NC5MI sample the minimum in the low temperature region almost disappears.

Such behavior of the M(T) curve may suggest that there are regions of different magnetic properties in the sample, which are revealed during the temperature change. Most likely, these are antiferromagnetic and ferromagnetic regions [14], the mutual volume ratio of which changes during temperature changes. Figure 2a shows that around 150 K, the material undergoes a phase change followed by a further increase in magnetization, meaning that the ferromagnetic region grows at the expense of another one.

Comparing this nature of the change in magnetization as a function of temperature with the results of measurement of the magnetization of a sample with the same composition but not subjected to annealing, it can be concluded that the annealing causes a change in the structure of the Heusler alloy. The authors of [14] report on the phase separation phenomenon in the ${\mathrm{Ni}}_{50}{\mathrm{Mn}}_{45}{\mathrm{In}}_{5}$ alloy during annealing in the presence of a magnetic field. In our case, annealing took place without an external field and the composition of the sample was different, containing more indium and less manganese. It seems, however, that the annealing process could also in our case cause a change in the structure, i.e., precipitation of various phases of the alloy. Indirect evidence for this may be the composition measurements carried out for the surfaces of the annealed samples, which reveal a different percentage of elements in relation to the unheated samples [20]. The comparison of Figure 2a–c shows that increasing the cobalt concentration causes homogeneity of these areas, i.e., the structural heterogeneity in the volume of the alloy decreases.

Another explanation of the observed properties can be based on [23], the authors of which report a similar relationship M(T) of ${\mathrm{Ni}}_{2.14}{\mathrm{Mn}}_{0.55}{\mathrm{Sb}}_{1.31}$ samples subjected to annealing at the temperature of 850 ${}^{\circ }$C. They assume that the crystal structure of the samples is the same in the whole volume, but the elements that compose it may be in different places in different areas. This can lead to magnetic interactions of the FM and AFM type, which compete with each other at different temperatures, resulting in the observed magnetization.

**Figure 3:**
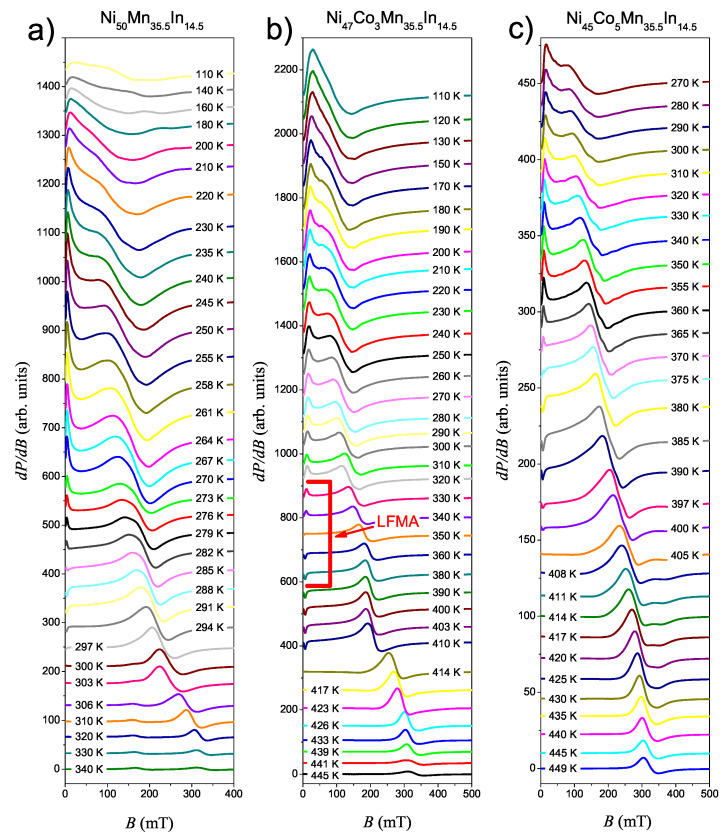
Selected EMR spectra of derivative of the resonance absorption (dP/dB) for ${\mathrm{Ni}}_{50-x}{\mathrm{Co}}_{x}{\mathrm{Mn}}_{35.5}{\mathrm{In}}_{14.5}$ ribbons: ${\mathrm{Ni}}_{50}{\mathrm{Mn}}_{35.5}{\mathrm{In}}_{14.5}r$ (**a**), ${\mathrm{Ni}}_{47}{\mathrm{Co}}_{3}{\mathrm{Mn}}_{35.5}{\mathrm{In}}_{14.5}$ (**b**) and ${\mathrm{Ni}}_{45}{\mathrm{Co}}_{5}{\mathrm{Mn}}_{35.5}{\mathrm{In}}_{14.5}$ (**c**) registered during a cooling process. Spectra are vertically shifted for clarity. The example of the LFMA signal is marked in red.

Figure 3 displays some selected EMR spectra for ${\mathrm{Ni}}_{50-x}{\mathrm{Co}}_{x}{\mathrm{Mn}}_{35.5}{\mathrm{In}}_{14.5}$ ribbons with different concentrations of Co atoms. The spectra were registered within the temperature range of 110 K ≤T≤ 450 K. In general, the annealing process does not drastically change the character of the EMR signal registered for ${\mathrm{Ni}}_{50-x}{\mathrm{Co}}_{x}{\mathrm{Mn}}_{35.5}{\mathrm{In}}_{14.5}$ [20,21]. At the highest temperatures, the main line has an unchanged asymmetric character. Such an observation confirms that the ribbons before annealing were already partially ordered materials.

At the highest temperatures the EMR spectra of NC0MI ribbon include two separated Dyson lines: very weak line 1 ($B_{R}∼330$ mT) and line 2 ($B_{R}∼200$ mT). Within the temperature range 400 K ≤T≤ 450 K, the amplitude of the line 1 is so weak that its contribution to the EMR signal can be neglected. Below 400 K, the amplitude of line 1 gradually increases, and at a temperature of about 345 K, line 1 reaches a height equal line 2. With the further reduction in temperature, the intensity of line 1 increases and this line shifts towards weaker fields, and in 300 K it overlaps with line 2. For this sample, the mainline (line 1) is similar in nature to the paramagnetic lines observed for the Ni-Mn-In ribbons in previous work [20,24].

The spectra of the sample NC3MI are presented in Figure 3b. In the temperature range 426 K ≤T≤ 445 K, a single strong, asymmetric line is observed. This is a typical paramagnetic line. Below 426 K EMR line starts broadening and shifts toward a low-field region. Figure 3c shows the EMR spectra for NC5MI registered within the temperature range of 270 K ≤T≤ 450 K. At the highest temperatures in the EMR spectrum, a single asymmetrical Dysonian line (line 1) is visible. In the range of temperatures 430 K ≤T≤ 450 K, the position of the line slightly changes. During cooling of the sample, below 425 K the main line shifts towards the low field region, and, additionally, in the EMR spectrum around 340 mT a much weaker symmetrical line appears (line 2). As the temperature decreases these two lines become better separated.

The resonance conditions below the magnetic phase transition temperature in ferromagnetic materials can be considered within the classical theory of ferromagnetic resonance proposed by Kittel [25]. The thickness of the ribbon (parallel to *x*-axis) is far smaller than other sample dimensions and we can treat it, in the first approximation, as a plane surface, where the magnetic field is parallel to it. This model corresponds to a special case of the Kittel equation, where the demagnetization factors are equal to $N_{x}=1$ and $N_{y}=N_{z}=0$, and the Kittel equation will be simplified considerably (SI units):

(1) $$\omega_{0}=\gamma {\left[(\mu_{0}M+B)B\right]}^{1/2},$$

where $\omega_{0}$ is the microwave angular frequency ($\omega_{0}=2\pi f_{0}$), γ is the gyromagnetic ratio, *B* means the static magnetic field, $\mu_{0}$ stands for the permeability, and *M* denotes the saturation magnetization. A Typical continuous wave EMR experiment has a fixed microwave frequency, solving the Equation (1) for *M*, assuming *B* as the value of the magnetic field corresponding to the resonance condition.

**Figure 4:**
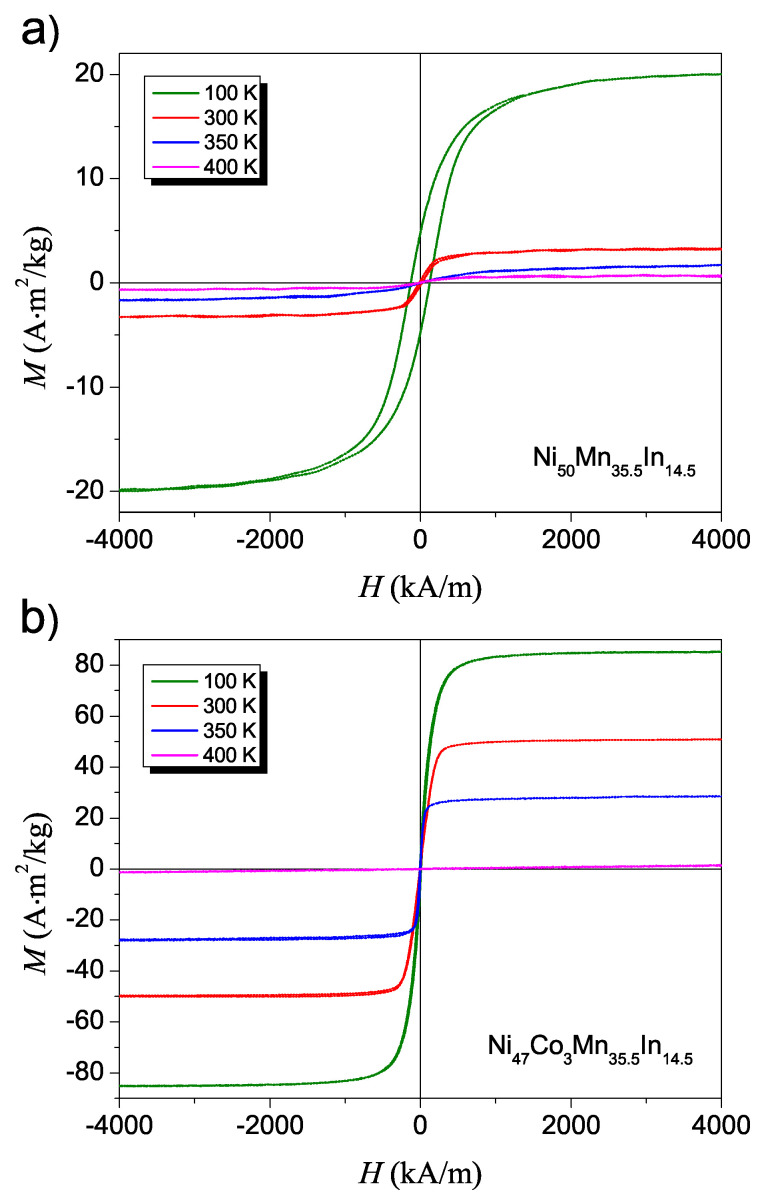
The field dependence of magnetization for ${\mathrm{Ni}}_{50}{\mathrm{Mn}}_{35.5}{\mathrm{In}}_{14.5}$ (**a**) and ${\mathrm{Ni}}_{47}{\mathrm{Co}}_{3}{\mathrm{Mn}}_{35.5}{\mathrm{In}}_{14.5}$ (**b**) ribbons at the selected temperatures.

Table 1 lists the *M* values calculated based on Equation (1) and determined from the hysteresis loops for particular temperatures. The latter value of magnetization *M* for each temperature was obtained from M(H) dependence presented in Figure 4. As can be seen for the NC3MI ribbon, the magnetization value *M* determined from Equation (1) is slightly higher than that from the figure. This difference can be related to the fact that EMR probes only the surface of a sample (∼1μm), while the VSM measurements provide information about bulk magnetization. Thus, in the EMR results, the outer material layer is taken into account, where the interaction of a ferromagnetic nature is dominant, while VSM measures the magnetization of the entire sample volume, thus the total magnetization may be reduced as a result of the presence of a different magnetic ordering.

At temperatures below $T_{C}$, in the EMR spectra registered for each sample, an additional signal appears, known in the literature as low-field microwave absorption (LFMA). Generally, the LFMA is observed for soft ferromagnetic materials, such as manganites, amorphous ribbons, magnetic thin films, magnetic nanoparticles [26,27,28,29,30]. In addition, LFMA is also used to detect ferromagnetism and to determine $T_{C}$. Furthermore, the absence of LFMA in the soft ferromagnetic sample is a good indicaton of existence of superparamagnetic state [29,30].

In our prior works, we observed LFMA in the Ni-Mn-In and Ni-Co-Mn-In ribbons [22,24] but the behaviour of LFMA in annealed ribbons is more complex. Below $T_{C}$ we observe only the fragment of the LFMA signal, but this signal has the same phase as the main line. With the further reduction in temperature, the LFMA signal changes their phase. The change in the signal phase for the sample without cobalt occurs at a temperature of approximately 291 K, for the sample with the cobalt content of 3 at.%, this temperature is higher, approx. 351 K. For the sample with the highest cobalt content (5 at.%), the phase change temperature is approximately 370 K. An especially interesting behavior of the LFMA can be observed for the NC3MI ribbon. The Curie temperature determined from VSM data for sample NC3MI is equal to 364 K (see Figure 2b), while the value of $T_{C}$ determined from the temperature dependence of the integrated intensity of EMR line is equal 421 K (see Table 2). The difference in the value of Curie temperature obtained from VSM and EMR is a consequence of the different natures of both techniques. The EMR is sensitive to changes in the environment of the magnetic ions, so it is a good method for detecting local changes in properties of materials [21]. For conducting samples, due to an existing skin depth phenomenon, and the local character of the EMR method, one can notice a discrepancy between results determined from EMR data and VSM technique. In our prior measurements, however, for as-spun ${\mathrm{Ni}}_{45}{\mathrm{Co}}_{5}{\mathrm{Mn}}_{35.5}{\mathrm{In}}_{14.5}$ [21] and annealed ${\mathrm{Ni}}_{50}{\mathrm{Mn}}_{35.5}{\mathrm{In}}_{14.5}$ [24], only a slight difference in $T_{C}$ values determined by using the EMR and VSM technique was observed, although for those samples the skin depth effect also exists. The difference in the Curie temperature values determined in both methods for samples described in this report may be explained by the annealing process, which caused the inhomogeneity of the samples. Ferromagnetic structures may have formed on the sample surface, which is manifested by the Curie 421 K temperature determined by the EMR method and the differences in the magnetization values calculated using the EMR and VSM techniques and those listed in Table 1. The sample taken as a whole, neglecting the thin layer near the surface, remains paramagnetic at this temperature, as indicated by M(T) and M(H) dependences. Ferromagnetism in the macro scale is revealed at a lower temperature, about 360 K, which is visible both in the magnetization data and in the anomalous behaviour of the EMR line, suggesting the occurrence of a phase transition.

**Figure 5:**
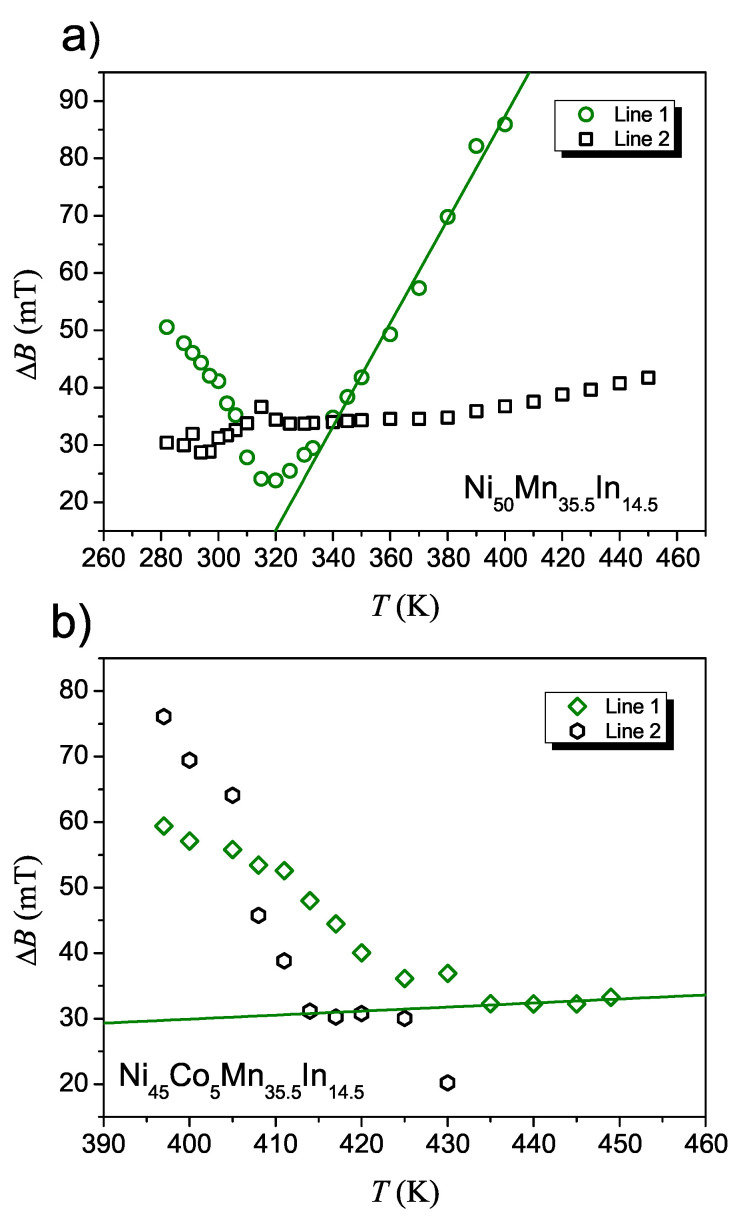
Temperature dependence of the EMR linewidth ΔB for ${\mathrm{Ni}}_{50}{\mathrm{Mn}}_{35.5}{\mathrm{In}}_{14.5}r$ (**a**) and ${\mathrm{Ni}}_{45}{\mathrm{Co}}_{5}{\mathrm{Mn}}_{35.5}{\mathrm{In}}_{14.5}$ (**b**) ribbons. The solid lines represent the best fit of the linewidth above $T_{C}$, using the expression (1).

Figure 5 exhibits the temperature dependence of linewidth $\Delta B\left(T\right)$ for NC0MI and NC5MI. In the temperature range of 280 K ≤T≤ 450 K the EMR spectra for NC0MI (Figure 5a) consists of two lines. In the whole temperature range, the ΔB of the line 2 varies slightly between 30–40 mT. In contrast, ΔB of line 1 in the temperature range of 330 K ≤T≤ 400 K decreases linearly, and then, after approaching Curie temperature, the linewidth starts to increase. This behaviour of $\Delta B\left(T\right)$ around $T_{C}$ is typical for ferromagnetic metals [31]. The high temperature EMR signal for NC5MI within temperature range 395 K ≤T≤ 450 K can be also divided into two lines, but the linewidth for NC5MI (Figure 5b) has a different character than the temperature dependence for NC0MI. The main line, which is labelled 1, shows linear behaviour of $\Delta B\left(T\right)$ only in a narrow range of temperature (430 K ≤T≤ 450 K) and its value is about 30 mT. Below these temperatures, the ΔB of both line increases. For NC3MI, no such relationship was determined due to the unregularly nature of changes in linewidth as a function of temperature.

The linear region of $\Delta B\left(T\right)$ observed in the temperature dependence for both samples, can be expressed by the Korringa relaxation model [32]:

(2) $$\Delta B\left(T\right)=a+b\cdot T,$$

where $a=\;\Delta B_{0}$ is the residual width and *b* is the Korringa rate.

The value of the last parameter *b*, describing the slope of $\Delta B\left(T\right)$, depends mainly on concentration of 3d elements, i.e., the *b* value increases when the number of 3d electrons decreases [33]. For NC0MI, the value of *b* is about 10 G/K (see Table 2) and this value is typically observed for transition metal EMR [34]. A similar value of this parameter was determined for the in-situ ribbon [20]. For the linear part of line 1 of NC5MI, the value of b is smaller and equal to 1 G/K. A similar value was observed in ${\mathrm{YBaMn}}_{2}O_{6}$ [35], where the bottleneck scenario for the spin relaxation of the manganese ion via mobile $e_{g}$ electron was noticed.

The main values determined from the EMR results, such as $T_{C}$, *a*, and *b* are collected in Table 2. Moreover, the table contains the EMR parameter values for in-situ [21,22] and another annealed ribbon [24] presented in our earlier works. The smallest changes in the EMR parameter values were observed for the sample without cobalt. However, in the paramagnetic regime, a weak additional line can be observed, which can be considered a singularity for Ni-Mn-In ribbons. Other parameters, including the Curie temperature, differ only by 2 K, which shows that the annealing parameters are not native, and the annealing itself significantly shifts the Curie temperature, i.e., for the in-situ ribbon, this temperature is 267 K [20] and it overlaps with the martensitic transformation temperature. It looks different in the case of NC3MI and NC5MI ribbons. For the sample with 3 at.% of cobalt the temperature dependence of $\Delta \left(B\right)$ has an irregular character and cannot be described using the Korringa relaxation model, while for as-cast ribbon with the same chemical composition, one can observe a typical Korringa relaxation process [22]. Furthermore, the Curie temperature, determined from the temperature dependence of the integral intensity, increases significantly relative to the non-annealed sample ($T_{C}=340$ K) [24] and differs from the Curie temperature for the NC3MI sample, determined from the temperature dependence of magnetization. In the case of the NC5MI ribbon, unlike the NC0MI ribbon and the non-annealed NC3MI ribbon, the width of the paramagnetic line (line 1) does not change significantly with temperature changes (above 430 K) and oscillates around the value ∼30 mT, and the parameters of the linear fit of the fragment $\Delta B\left(T\right)$ above $T_{C}$, equals a≈0 mT, and b=1 G/K. Such parameter values and the shape of $\Delta B\left(T\right)$ indicate that the temperature dependence of the line width for the annealed NC5MI ribbon cannot be described by the Korring relaxation model.

## 4. Conclusions

The effect of cobalt content of annealed ${\mathrm{Ni}}_{50-x}{\mathrm{Co}}_{x}{\mathrm{Mn}}_{35.5}{\mathrm{In}}_{14.5}$ for (x=0,3,5) is investigated through scanning electron microscopy, electron magnetic resonance, and vibrating sample magnetometer. The main conclusions can be listed as follows:

- The EMR and VSM results confirm that the annealing process shifts the magnetic phase transition characteristic temperatures. Furthermore, VSM measurement shows the existence of an additional magnetic phase transition below $T_{C}$.
- Comparing the EMR spectra for as-cast ribbons, published in our prior works, with results for annealed ribbons presented in this work we observe changes. The annealing caused the existence of an addition line in the paramagnetic regime for NC0MI and NC5MI samples.
- The differences in the Curie temperature determined by two methods (VSM and EMR) for the annealed NC3MI sample suggest that it is magnetically inhomogeneous, i.e., the subsurface layers have different magnetic properties than the interior of the sample.
- The scanning electron microscopy results show that the annealing process modifies the morphology of the samples with cobalt content. For ribbon without cobalt, the changes are not observed.
- The EMR spectra for all ribbons, registered below Curie temperature, contain the LFMA signal. During the cooling process, the LFMA signal changes its phase to the opposite of the main EMR signal.

## Figures and Tables

**Table 1:** The magnetization value determined from the solution of the Kittel equation (*M*) and from hysteresis loops ($M^{*}$) for NC0MI and NC3MI samples.

Label	*T* (K)	*M* (kA/m)	$M^{*}$ (kA/m)
NC0MI	300	147	26
	100	608	157
NC3MI	350	330	242
	300	553	416
	100	879	694

**Table 2:** The corresponding EMR results of NC0MI, NC3MI, and NC5MI samples and comparison with literature data. $T_{C}$ are determined from the temperature dependence of integral intensity, *a* and *b* parameters are determined from temperature dependence of the EMR linewidth.

Label	Composition	$T_{C}$ (K)	*a* (mT)	*b* (mT/K)
NC0MI	${\mathrm{Ni}}_{50}{\mathrm{Mn}}_{35.5}{\mathrm{In}}_{14.5}$	300	−260	0.87
[24] *	${\mathrm{Ni}}_{50}{\mathrm{Mn}}_{35.5}{\mathrm{In}}_{14.5}$	298	−220	0.8
NC3MI	${\mathrm{Ni}}_{47}{\mathrm{Co}}_{3}{\mathrm{Mn}}_{35.5}{\mathrm{In}}_{14.5}$	421	-	-
[22] **	${\mathrm{Ni}}_{47}{\mathrm{Co}}_{3}{\mathrm{Mn}}_{35.5}{\mathrm{In}}_{14.5}$	340	−244	0.77
NC5MI	${\mathrm{Ni}}_{45}{\mathrm{Co}}_{5}{\mathrm{Mn}}_{35.5}{\mathrm{In}}_{14.5}$	414	−9	0.1

* annealed at 600 K; ** non-annealed.

## Data Availability

Not applicable.
